# Correction: Jin et al. Comparative Evaluation of STEAP1 Targeting Chimeric Antigen Receptors with Different Costimulatory Domains and Spacers. *Int. J. Mol. Sci.* 2024, *25*, 586

**DOI:** 10.3390/ijms251810024

**Published:** 2024-09-18

**Authors:** Yixin Jin, Claire Dunn, Irene Persiconi, Adam Sike, Gjertrud Skorstad, Carole Beck, Jon Amund Kyte

**Affiliations:** 1 Department of Cancer Immunology, Institute for Cancer Research, Oslo University Hospital, 0379 Oslo, Norway; yixin.jin@rr-research.no (Y.J.); claire.dunn@ous-research.no (C.D.); irene.persiconi@rr-research.no (I.P.); adam.sike@ous-research.no (A.S.); c.a.beck@ous-research.no (C.B.); 2 Department of Clinical Cancer Research, Oslo University Hospital, 0424 Oslo, Norway; 3 Faculty of Health Sciences, Oslo Metropolitan University, 0130 Oslo, Norway

In the original publication [1], there was a mistake in Figure 6A. When the IVIS images of two mice in JK10 group day 17 were cropped from the original larger image to fit with the layout, a mistake occurred so that one JK10 mouse was replaced with a JK11 mouse. The analyzed data were correct; as were Figure 6B–D. Only the image of the individual mouse was wrong. The corrected Figure 6 appears below. The authors state that the scientific conclusions are unaffected. This correction was approved by the Academic Editor. The original publication has also been updated.

## Figures and Tables

**Figure 6:**
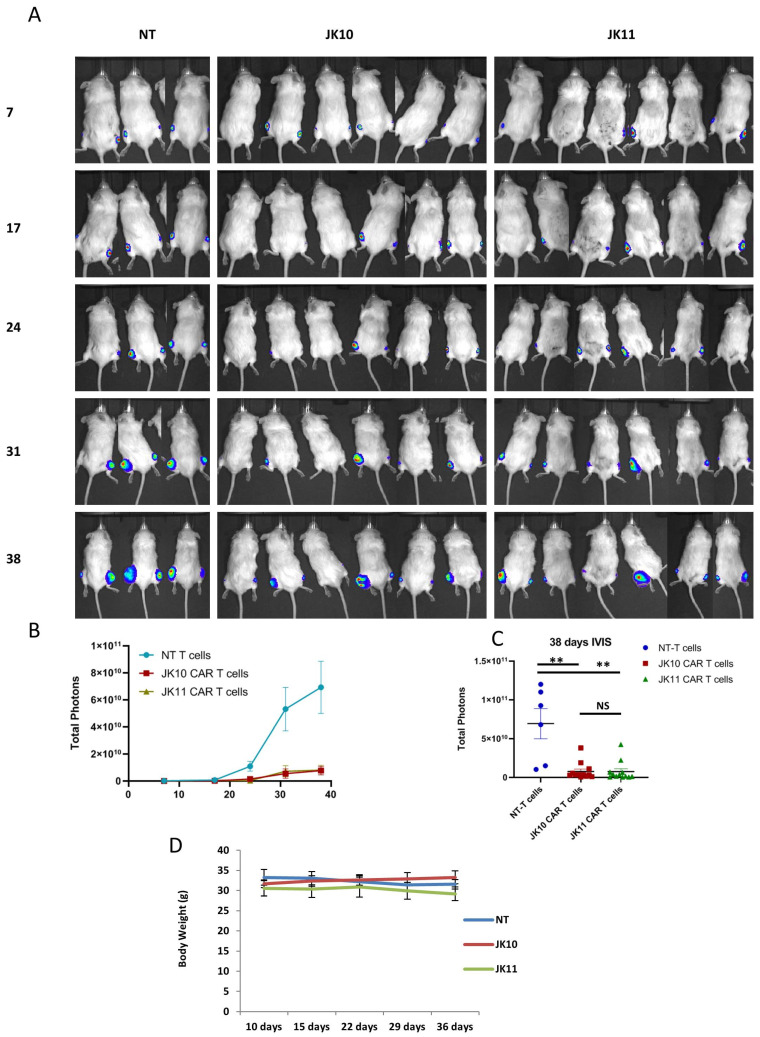
CD28 and 4-1BB CAR T cells inhibit tumor growth in vivo. (**A**) Bioluminescence images of individual mice at multiple time points in a subcutaneous tumor model. NSG mice were engrafted subcutaneously on both hind legs with 2 × 10^6^ luciferase expressing 22Rv1 prostate cancer cells. On days 10 and 17, the mice were treated with 1 × 10^7^ non-transduced (NT) T cells (N_tumor_ = 6) or CD28 CAR T cells (N_tumor_ = 12) and 4-1BB CAR T cells (N_tumor_ = 12) by intravenous injection. Bioluminescence signals of individual mice were measured at indicated time points (days after tumor engraftment). (**B**) Tumor growth is measured once a week by bioluminescence IVIS imaging. The data are presented as the mean ± SEM of tumors in each group. (**C**) Bioluminescence signals (mean ± SEM) 38 days after tumor engraftment. Statistical analyses in (**B**,**C**) were performed with the Mann–Whitney U test. NS (not significant), ** *p* < 0.001. (**D**) The body weight of the mice was measured once per week after the first treatment with T cells on day 10. Error bars indicate SD.
