# Supplementary material for: Comparative Evaluation of STEAP1 Targeting Chimeric Antigen Receptors with Different Costimulatory Domains and Spacers
Source: Int J Mol Sci. 2024 Jan 2;25(1):586. doi: 10.3390/ijms25010586 (PMC10778617; doi:10.3390/ijms25010586)
Supplement: Supplementary file 1 [file ijms-25-00586-s001.zip › Figure S1.pptx]

## Slide 1
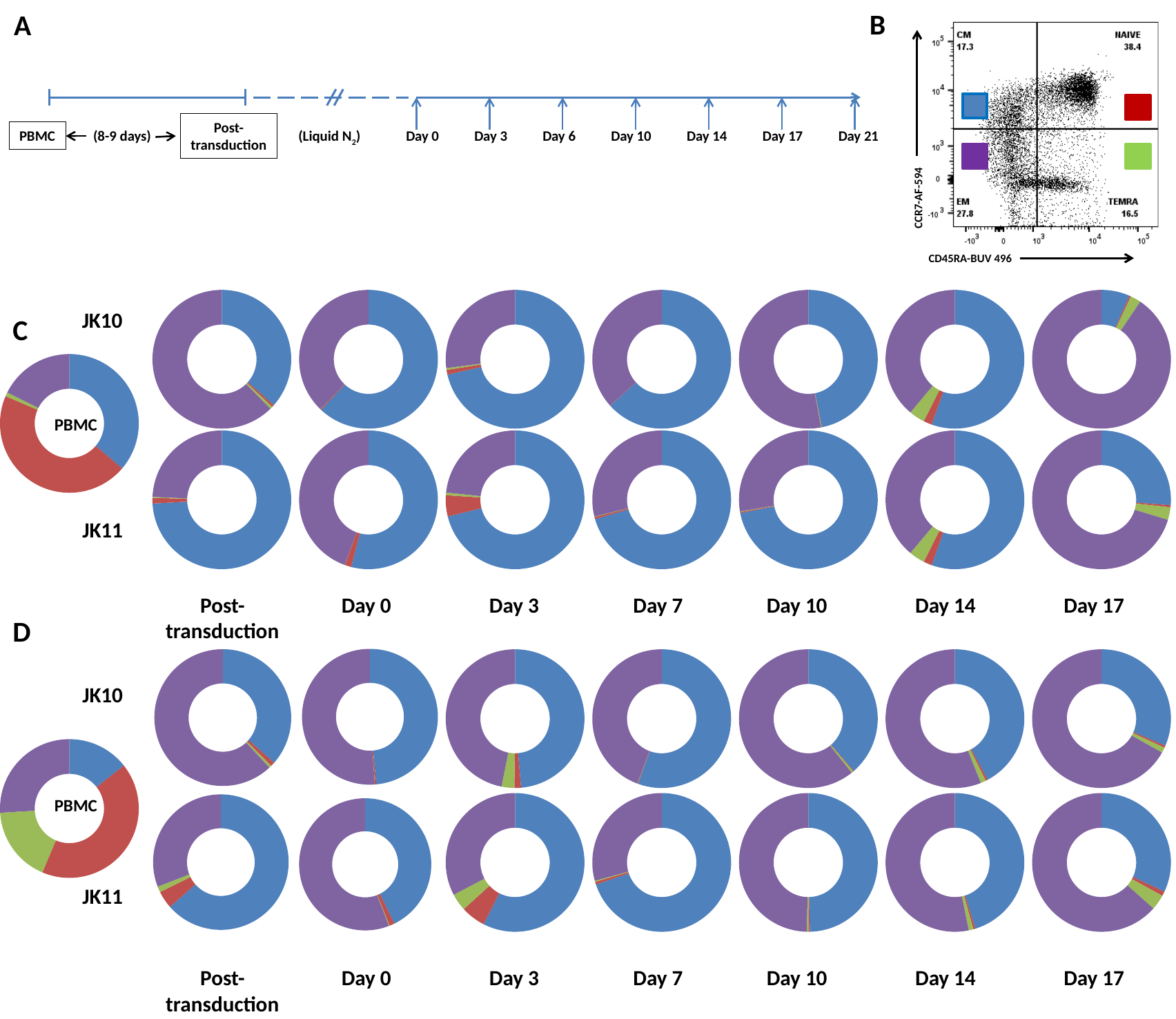

A
B
CCR7-AF-594
CD45RA-BUV 496
Post-
transduction
PBMC
(8-9 days)
(Liquid N2)
 Day 0
 Day 3
 Day 6
 Day 10
 Day 14
 Day 17
 Day 21
### Chart
| Category | |
|---|---|
### Chart
| Category | |
|---|---|
### Chart
| Category | |
|---|---|
### Chart
| Category | |
|---|---|
### Chart
| Category | |
|---|---|
### Chart
| Category | |
|---|---|
### Chart
| Category | |
|---|---|JK10
C
### Chart
| Category | |
|---|---|PBMC
### Chart
| Category | |
|---|---|
### Chart
| Category | |
|---|---|
### Chart
| Category | |
|---|---|
### Chart
| Category | |
|---|---|
### Chart
| Category | |
|---|---|
### Chart
| Category | |
|---|---|
### Chart
| Category | |
|---|---|JK11
Post-
transduction
Day 0
Day 3
Day 7
Day 10
Day 14
Day 17
D
### Chart
| Category | |
|---|---|
| CM | 36.48333333333333 |
| Naive | 0.9950000000000001 |
| TEMRA | 0.5783333333333337 |
| EM | 61.93333333333334 |
### Chart
| Category | |
|---|---|
### Chart
| Category | |
|---|---|
### Chart
| Category | |
|---|---|
### Chart
| Category | |
|---|---|
### Chart
| Category | |
|---|---|
### Chart
| Category | |
|---|---|JK10
### Chart
| Category | |
|---|---|
| CM | 14.393333333333334 |
| Naive | 41.98333333333334 |
| TEMRA | 17.63333333333328 |
| EM | 26.0 |
### Chart
| Category | |
|---|---|
| CM | 63.51666666666647 |
| Naive | 4.186666666666667 |
| TEMRA | 1.3766666666666667 |
| EM | 30.899999999999988 |
### Chart
| Category | |
|---|---|
### Chart
| Category | |
|---|---|
### Chart
| Category | |
|---|---|
### Chart
| Category | |
|---|---|
### Chart
| Category | |
|---|---|
### Chart
| Category | |
|---|---|PBMC
JK11
Post-
transduction
Day 0
Day 3
Day 7
Day 10
Day 14
Day 17

## Slide 2
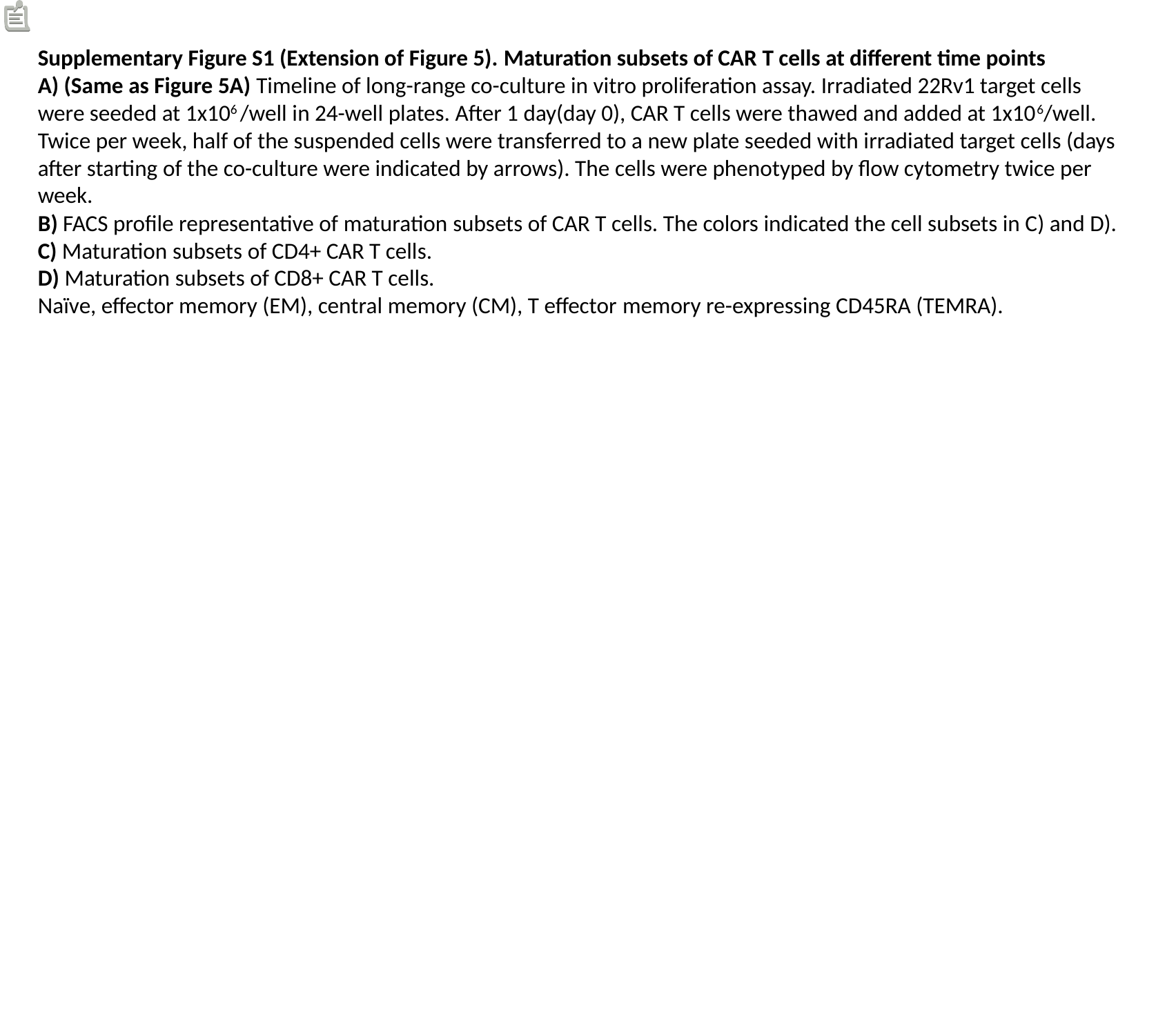

Supplementary Figure S1 (Extension of Figure 5). Maturation subsets of CAR T cells at different time points
A) (Same as Figure 5A) Timeline of long-range co-culture in vitro proliferation assay. Irradiated 22Rv1 target cells were seeded at 1x10^6^ /well in 24-well plates. After 1 day(day 0), CAR T cells were thawed and added at 1x10^6^/well. Twice per week, half of the suspended cells were transferred to a new plate seeded with irradiated target cells (days after starting of the co-culture were indicated by arrows). The cells were phenotyped by flow cytometry twice per week.
B) FACS profile representative of maturation subsets of CAR T cells. The colors indicated the cell subsets in C) and D).
C) Maturation subsets of CD4+ CAR T cells.
D) Maturation subsets of CD8+ CAR T cells.
Naїve, effector memory (EM), central memory (CM), T effector memory re-expressing CD45RA (TEMRA).
