# Supplementary material for: Comparative Evaluation of STEAP1 Targeting Chimeric Antigen Receptors with Different Costimulatory Domains and Spacers
Source: Int J Mol Sci. 2024 Jan 2;25(1):586. doi: 10.3390/ijms25010586 (PMC10778617; doi:10.3390/ijms25010586)
Supplement: Supplementary file 1 [file ijms-25-00586-s001.zip › Figure S2.pptx]

## Slide 1
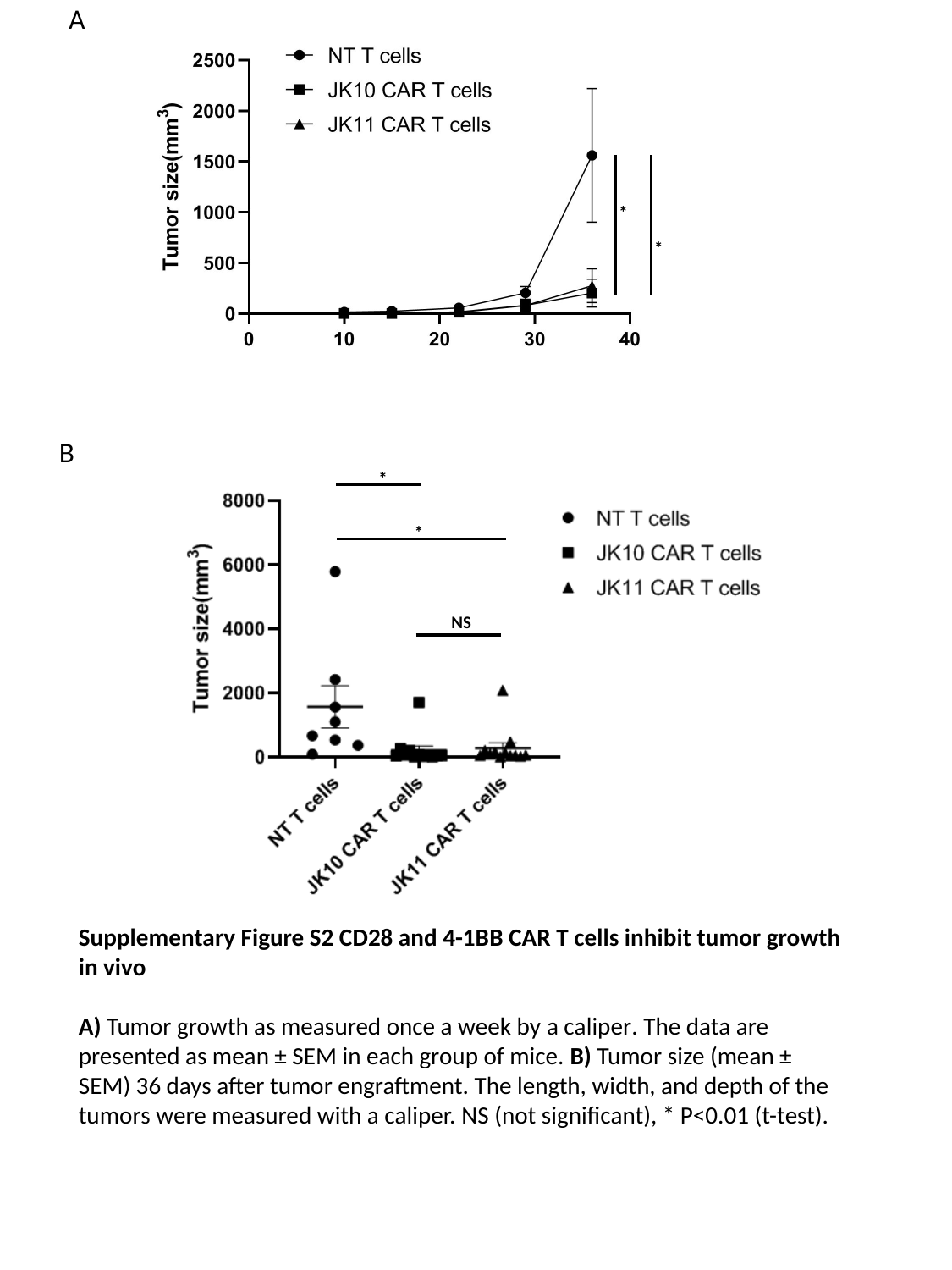

A
*
*
B
*
*
NS
Supplementary Figure S2 CD28 and 4-1BB CAR T cells inhibit tumor growth in vivo
A) Tumor growth as measured once a week by a caliper. The data are presented as mean ± SEM in each group of mice. B) Tumor size (mean ± SEM) 36 days after tumor engraftment. The length, width, and depth of the tumors were measured with a caliper. NS (not significant), * P<0.01 (t-test).
