# Supplementary material for: Comparative Evaluation of STEAP1 Targeting Chimeric Antigen Receptors with Different Costimulatory Domains and Spacers
Source: Int J Mol Sci. 2024 Jan 2;25(1):586. doi: 10.3390/ijms25010586 (PMC10778617; doi:10.3390/ijms25010586)
Supplement: Supplementary file 1 [file ijms-25-00586-s001.zip › Supplementary Table S1.pptx]

## Slide 1
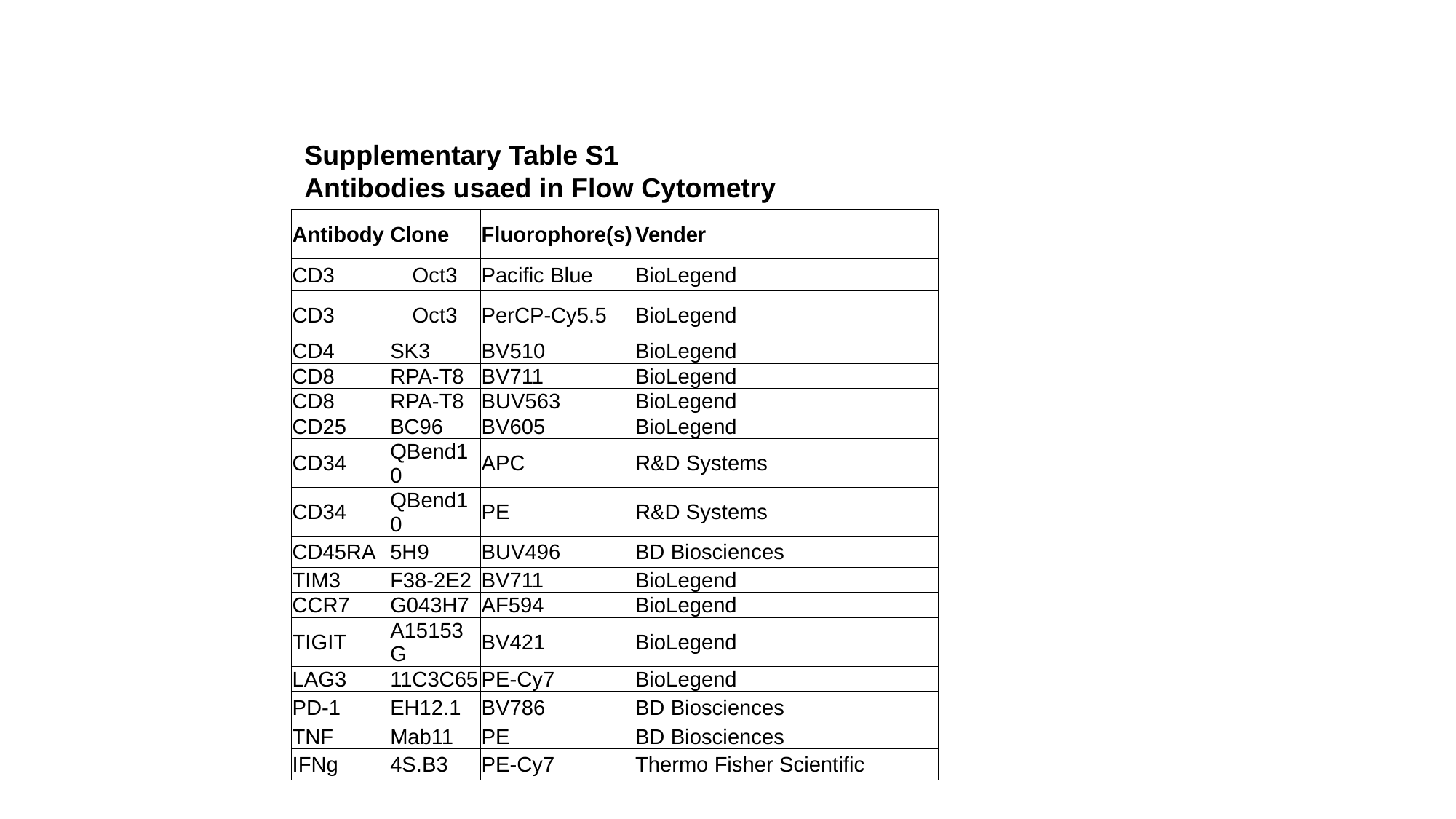

Supplementary Table S1
Antibodies usaed in Flow Cytometry
| Antibody | Clone | Fluorophore(s) | Vender |
| --- | --- | --- | --- |
| CD3 | Oct3 | Pacific Blue | BioLegend |
| CD3 | Oct3 | PerCP-Cy5.5 | BioLegend |
| CD4 | SK3 | BV510 | BioLegend |
| CD8 | RPA-T8 | BV711 | BioLegend |
| CD8 | RPA-T8 | BUV563 | BioLegend |
| CD25 | BC96 | BV605 | BioLegend |
| CD34 | QBend10 | APC | R&D Systems |
| CD34 | QBend10 | PE | R&D Systems |
| CD45RA | 5H9 | BUV496 | BD Biosciences |
| TIM3 | F38-2E2 | BV711 | BioLegend |
| CCR7 | G043H7 | AF594 | BioLegend |
| TIGIT | A15153G | BV421 | BioLegend |
| LAG3 | 11C3C65 | PE-Cy7 | BioLegend |
| PD-1 | EH12.1 | BV786 | BD Biosciences |
| TNF | Mab11 | PE | BD Biosciences |
| IFNg | 4S.B3 | PE-Cy7 | Thermo Fisher Scientific |
